# Insights for Fostering Resilience in Young Adults With Multiple Sclerosis in the Aftermath of the COVID-19 Emergency: An Italian Survey

**DOI:** 10.3389/fpsyt.2020.588275

**Published:** 2021-02-22

**Authors:** Valeria Donisi, Alberto Gajofatto, Maria Angela Mazzi, Francesca Gobbin, Isolde Martina Busch, Annamaria Ghellere, Michela Rimondini

**Affiliations:** ^1^Section of Clinical Psychology, Department of Neuroscience, Biomedicine and Movement Sciences, University of Verona, Verona, Italy; ^2^Section of Neurology, Department of Neuroscience, Biomedicine and Movement Sciences, University of Verona, Verona, Italy

**Keywords:** pandemic, COVID-19, multiple sclerosis, resilience, psychological adjustment, coping strategies, psychological support

## Abstract

**Objective:** Recent evidence has demonstrated that the COVID-19 pandemic is taking a toll on the mental health of the general population. The psychological consequences might be even more severe for patients with special healthcare needs and psychological vulnerabilities due to chronic diseases, such as multiple sclerosis (MS). Thus, we aimed to explore the psychological impact of this pandemic and of the subsequent healthcare service changes on young adults with MS living in Italy and to examine their coping strategies and preferences regarding psychological support in the aftermath of the pandemic.

**Methods:** Data were collected using a cross-sectional, web-based survey advertised on social networks. We report both quantitative (descriptive statistics, *t*-tests, and one-way ANOVA) and qualitative data (inductive content analysis).

**Results:** Two hundred and forty-seven respondents (mean age 32 ± 7 years), mainly with relapsing–remitting MS, from all Italian regions participated. Participants felt more worried, confused, sad, and vulnerable because of the disease “during” the pandemic in comparison to their self-evaluation of the period “before” the COVID-19 outbreak. Similarly, their perception of control over MS decreased “during” the pandemic in comparison to the retrospective evaluation of the period “before” the COVID-19 outbreak (*p* < 0.01). Canceled/postponed visits/exams were listed as the most frequent MS management changes, with modified/postponed pharmacological treatment representing the most stressful change. Psychological support in dealing with pandemic-related fears and improving MS acceptance and well-being was considered extremely important by almost 40% of the respondents. Different coping strategies were mentioned in the qualitative section of the survey, with social support, hobbies, and keeping busy being the most frequent ones.

**Conclusions:** Considering the enormous impact of the pandemic on young adults with MS, we urge MS clinical centers to implement psychological support programs that address the potentially long-lasting psychological negative impact, thus fostering the therapeutic alliance that is being threatened by the infection prevention measures imposed during the pandemic, and promoting psychological resources for adaptively managing future waves of COVID-19.

## Introduction

Multiple sclerosis (MS) is the most common chronic neurological disease causing disability in young adults. Relapsing forms are characterized by acute/subacute onset of neurological symptoms followed by complete or partial recovery and subsequent periods of relative well-being, whereas in the progressive forms, the disease shows a worsening of neurological symptoms with an increase in disability independent of relapses (1). In addition to the possible physical limitations due to neurological symptoms, MS patients experience psychological distress and are at higher risk of depression and anxiety compared to the general population (2–4). Since MS is generally diagnosed in a stage of life of great significance for the achievement of personal goals (i.e., between the ages of 20 and 40) (5, 6), the adaptation to this chronic disease, especially in the first years after MS diagnosis, may become even more emotionally challenging (7–12).

It is widely recognized that MS pathogenesis is driven by an immune system dysregulation targeting the central nervous system (13, 14). For this reason, currently available disease-modifying therapies are represented by drugs with immunomodulatory and/or immunosuppressive mechanisms of action, which may significantly ameliorate the disease course (15, 16). While this is clearly reassuring for patients and clinicians, the potential risk of adverse events may be worrisome, particularly in situations of vulnerability. In routine clinical practice of many countries, patients with MS regularly access the outpatient clinic both to check their clinical status and to monitor the effectiveness and safety of ongoing treatment; the frequency of visits depends on patient age and disability, disease characteristics, and therapy. Typically, each MS patient contacts or accesses a specialized clinical center multiple times a year (17).

In December 2019, a respiratory infection (i.e., COVID-19) caused by a novel coronavirus, namely, severe acute respiratory syndrome coronavirus 2 (SARS-CoV-2), was detected in China and rapidly spread worldwide in the following weeks, causing soon a pandemic (18). Italy has been one of the first and most severely affected countries outside China (239,410 infected people with 34,644 deaths as of June 24 2020—Istituto Superiore di Sanità) (19), leading the Italian government to initially declare the state of emergency starting from January 31, 2020, and, subsequently, from the end of February, to implement progressive restrictions of movement culminating in the country lockdown imposed from March 10 to May 3 2020, followed by a gradual lifting of these measures in the months of May and June 2020. The emergency due to the COVID-19 pandemic has significantly impeded the regular access of patients to MS centers. Indeed, among other restrictions, the health security measures taken by the Italian Government to contain the spread of infection imposed the suspension of non-urgent care (20). To ensure continuity of care and treatment for MS patients, clinicians opted for alternative communication strategies, such as telemedicine tools (e.g., emails, phone calls, and videocalls) (21–23).

Recent studies have highlighted the psychological burden of the COVID-19 pandemic, including high prevalence rates of psychological symptoms and disorders, with potentially long-lasting effects (24–26). As indicated by previous experience during the 2002/2003 severe acute respiratory syndrome coronavirus epidemic, psychological interventions could be a favorable option also for chronic patients to deal with the adverse psychological impact (27). However, to the best of our knowledge and at the moment of preparation of the current paper, studies exploring the psychological impact of the COVID-19 pandemic on patients with MS are still sparse. Those articles do not explore specifically the changes in patients' disease perceptions or expectations on psychological support (28–33). Moreover, none of these articles integrated quantitative and qualitative methods.

To fill this gap, the aims of this explorative study were 4-fold: (i) to describe the potential psychological impact of the COVID-19 pandemic on MS perceptions of young adults with MS, (ii) to examine changes in the management of the disease and the provision of health services and their perceived stress linked to those changes, (iii) to explore their preferences in terms of psychological support, and (iv) to explore their coping strategies in facing the pandemic consequences.

To achieve these aims, qualitative and quantitative methods have been synergically applied. In particular, a quantitative approach has been applied for the first three aims and a qualitative one for the latter. We assumed an increase of negative emotions regarding MS and of the sense of vulnerability and a reduction in control perception over MS due to the pandemic, a high level of perceived stress linked to the changes in the management of MS regarding different aspects of MS care. Further, we supposed that young adults with MS would consider psychological support highly relevant.

## Methods

The study is part of a larger project (ESPRIMO project), a prospective program aiming to study and promote resilience in young adults with MS. This general objective will be pursued through the activation of a working group that will involve, in the different phases of the program, the main stakeholders: health professionals (e.g., neurologists, psychologists, nurses, and rehabilitators), researchers from multiple disciplines related to basic research and applied research, MS patient associations (e.g., local Italian Multiple Sclerosis Association), patients with MS, territorial bodies (e.g., municipality), and private entities (e.g., foundations). The team is coordinated by a group of clinicians from the Regional Multiple Sclerosis Center of the Borgo Roma Hospital of the Integrated University Hospital of Verona and researchers from the Department of Neuroscience, Biomedicine and Movement of the University of Verona, engaged in care and research activity in the field of MS for many years. The final goal of the project is to develop and evaluate the effectiveness of a biopsychosocial intervention that promotes resilience and adaptation to the disease to increase quality of life in young adults with MS. The project was activated in 2018 as part of the departmental development program “Behavior and well-being: a multidisciplinary approach to promote the quality of life in conditions of vulnerability” of the Department of Neuroscience, Biomedicine and Movement of the University of Verona funded by the Ministry of Education, University and Research. The two core elements of the ESPRIMO project are the BPS-ARMS study and the ESPRIMO feasibility study. The first study aims to explore the resilience and quality of life of young patients by adopting a biopsychosocial approach, that is, by studying the possible clinical, biological, social, and psychological factors connected to adaptation and resilient reaction to disease event in patients at onset. The second study will last 24 months, following three main consequential phases: the initial co-creation phase aiming to develop a biopsychosocial intervention (the so-called ESPRIMO intervention) targeted at young adults with MS; the intervention phase aiming to test preliminary effect, feasibility, and acceptability of the ESPRIMO intervention in a sample of young patients with MS; and the third phase aiming to fine-tune the ESPRIMO intervention.

As part of the project “ESPRIMO,” the present study has been approved by the Ethical Committee of the Verona Hospital (Prog. 2676CESC) and registered on ClinicalTrials (ClinicalTrials.govID: NCT04431323). The study uses a cross-sectional, observational design and followed the STROBE checklist (34).

Data were collected between May 13 and June 3, 2020, using a web-based, anonymous survey. Young adults with MS, being a resident of Italy during the COVID-19 pandemic, and meeting the following inclusion criteria could participate: age 18–45 years, MS diagnosis, Italian speaker, and electronic informed consent signed.

According to the ESPRIMO project, the definition of the age range for “young adults” from 18 to 45 years is made on the basis of the clinical onset of MS and course of the disease. Indeed, it has to be noticed that a clear age cut-off for the definition of “young adult” has not been established in the medical field, and, as discussed in a previous paper in the field of neurology in Italy, an age cut-off might be considered arbitrary (35). In our case, the age range has been defined on the basis of the age range of MS onset (i.e., 20–40 years), extending the age of inclusion, setting the minimum age to 18, and widening the maximum age to 45. MS is a chronic disease and typically long clinical course leading to a relevant group of patients reaching elderly with the disease. Consistently, Garcia and Finlayson (36) defined people from the age of 45 as “aging with MS”. Therefore, we focused on a subgroup of MS patients who could be considered “young” given the disease history.

The survey was advertised on the Facebook page and the Instagram profile of the ESPRIMO project as well as in several Facebook groups focusing on MS. The advertisement also encouraged people to share the survey link to others who are potentially eligible. Electronic informed consent was obtained from all participants prior to data collection.

### Survey

We created an *ad hoc*, self-administered questionnaire composed of closed and open questions, divided into four sections aiming to explore the following topics: psychological impact (section 1 and section 2b), changes in MS management (section 2a), preferences regarding psychological support (section 3), and psychological resources (section 4) (Appendix 1). Moreover, some sociodemographic and clinical data have been collected.

The psychological impact of COVID-19 has been quantitatively explored in section 1: self-reported perceptions about the MS regarding emotions, illness perception, and commitment to deal with MS before and during the pandemic. Since the existing brief and validated questionnaires were not able to explore all these specific areas, we created an *ad hoc* scale, composed of seven items evaluating the following MS perceptions on two 10-point Likert scales (i.e., before the COVID-19 emergency and during the COVID-19 emergency): a range of feelings about the MS disease (i.e., anxiety, confusion, sadness), illness perceptions (i.e., vulnerability, control over illness), and commitment to deal with MS (i.e., commitment to manage the disease and to seek social support). The analysis of the Cronbach alpha considering these seven items indicates a high reliability for the two considered time frames (before COVID-19 = 0.81 and during COVID-19 = 0.84).

The second section explored both (a) changes in the services offered by clinical centers (e.g., medical visits, psychological visits, pharmacological treatment, and telemedicine) and (b) psychological impact in terms of perceived stress linked to the changes in MS care. When answering to this question, respondents could pick up more than one change and also add other changes not listed in the questionnaire. Stress was assessed using a 10-point Likert scale, ranging from 1 (“no stress”) to 10 (“maximum level of stress”).

Moreover, a third section explored needs and preferences regarding psychological support of young adults with MS.

Finally, the fourth section qualitatively investigated participants' psychological resources (namely: coping strategies, helpful/positive thoughts and learnings). In the present study, we will focus on coping strategies, defined by Lazarus and Folkman (37) as “constantly changing cognitive and behavioral efforts to manage specific external and/or internal demands that are appraised as taxing or exceeding the resources of the person”. Coping strategies were investigated by asking the following question: “*Please complete the following sentence: The strategy that has proven most useful for getting through this pandemic period has been...”*

The survey has been implemented using the software LimeSurvey, an open source online tool that allows to develop, publish, and collect responses to surveys.

### Data Analysis

#### Statistical Analysis

Descriptive statistics were presented as mean values and standard deviation (SD) for continuous variables and as absolute and relative frequencies for categorical variables. A set of Student's paired *t*-tests were applied to compare participants' levels of negative emotions, illness perception, and commitment to deal with MS before and during the pandemic. Student's two-group *t*-tests and one-way ANOVA were used to explore the differences across the main sociodemographic variables (see Tables 1–5 in Appendix 2; gender differences have been explored only on a sub-sample of participants).

A choropleth map, showing the spatial distribution of respondents among the Italian regions, was drawn with the Stata package grmap. All the analyses were performed with STATA 15.

#### Inductive Content Analysis

Participants' answers to the open question on coping strategies were grouped in an Excel file. Two researchers (AGh and VD) independently analyzed all answers and created a list of possible labels. Interrater reliability had good results: the percentage of agreement was 90% (CI 86–94%), Krippendorff's Alpha 0.88 (CI 0.84–0.93). These labels were then compared in a plenary meeting, and concordant and discordant labels were discussed with a third reviewer (MR). As a next step, all answers were coded using the finalized labels. Answers containing more than one type of coping strategy were divided into different utterances and coded separately. Frequencies of different types of coping strategies are reported.

## Results

### Sociodemographic Characteristics of the Sample

Of the 368 young adults with MS who accessed the survey, 67 were excluded according to the exclusion criteria (i.e., 14 for age, 53 not giving the consent). Furthermore, 52 of the surveys were returned empty or with only sociodemographic information compiled and 2 were compiled by residents abroad during the pandemic and thus excluded from further analysis, leaving a total sample of 247 respondents included in the analysis.

Respondents reported a mean age of 32 years (SD = 7) covering all possible ages (i.e., 18–45 years); 44% of respondents were 18–30 years old. Of all respondents, 46% were married or living with a partner, 55% were occupied, and 44% presented at least an academic degree.

**Figure 1:**
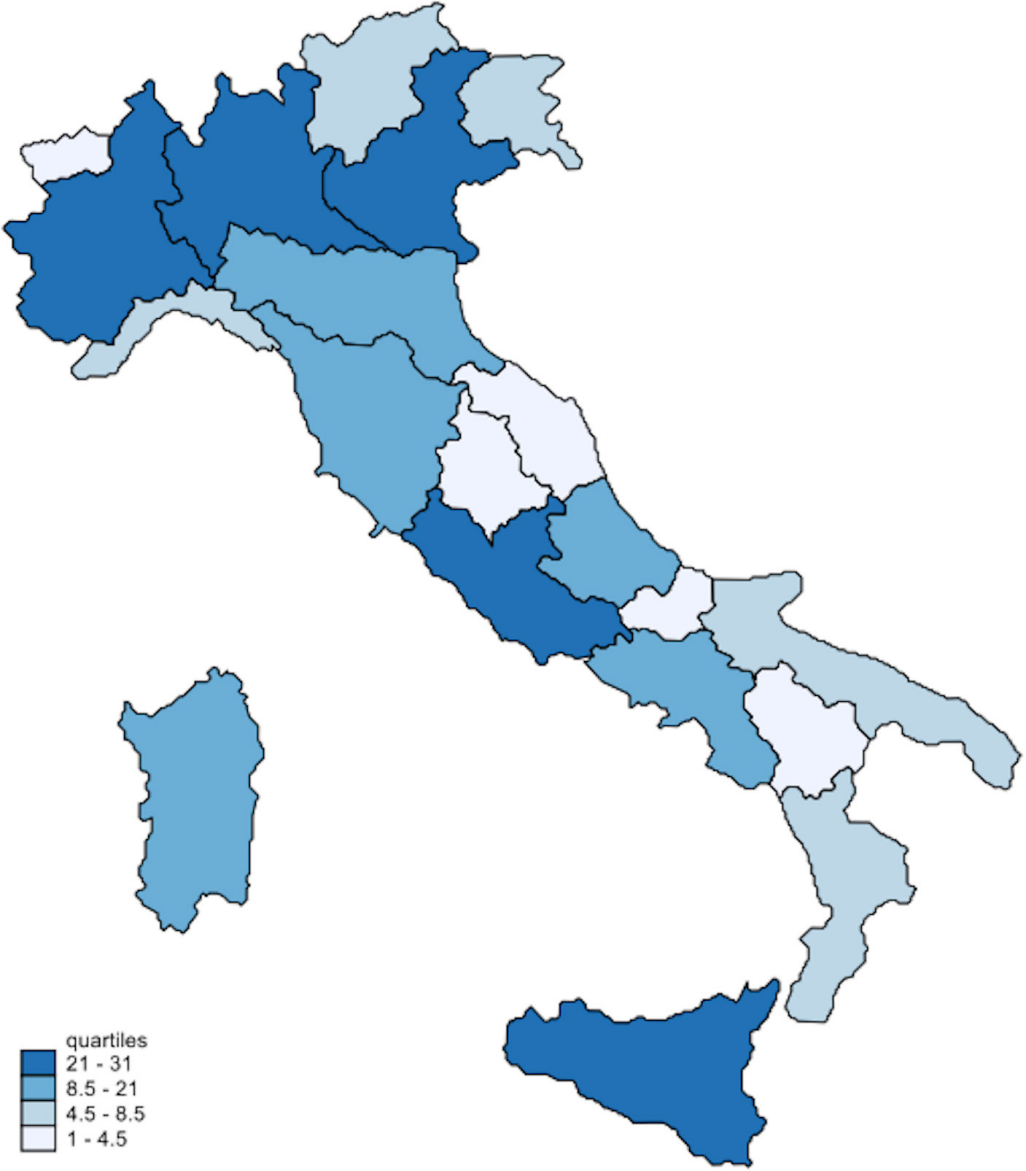
Map of the respondents' frequency distribution by regions (*n* = 247).

Regarding the place of residence during the COVID-19 pandemic, surveys were completed in all Italian regions, with 117 (47%) respondents living in the north, 47 (19%) in the center, and 83 (34%) in the south of the country (see Figure 1).

Most respondents reported a diagnosis of relapsing–remitting MS (227; 92%), whereas 7 (3%) had primary progressive MS, 8 (3%) had secondary progressive MS, and 5 (2%) had clinically isolated syndrome (CIS).

### Emotions Regarding MS, Illness Perceptions, and Commitment to Deal With the Disease Before and During the COVID-19 Pandemic

Table 1 summarizes patients' emotions, illness perceptions, and commitment to deal with the disease before and during the pandemic and the impact of COVID-19, calculated as the difference between the values expressed in the two phases (“during” vs. “before”). The mean levels of anxiety/worry and sadness/discouragement linked to MS significantly increased during the pandemic (*p* < 0.01). Similarly, respondents were significantly more confused/disoriented regarding their MS (*p* < 0.01). In addition, we found a negative impact of the pandemic on patients' illness perception, with a significant increase of vulnerability perception and a reduction of personal control over MS (*p* < 0.01). Conversely, patients were equally committed before and during the pandemic to find support and effective strategies to manage the disease.

**Table 1:** Self-reported perceptions about the MS: emotions, illness perception, and commitment to deal with MS before and during the pandemic (*n* = 247).

**Items**	**Before the COVID-19 pandemic (mean, DS)**	**During the COVID-19 pandemic (mean, DS)**	**Impact of COVID-19 pandemic (mean, DS)**	***t*-test**	**Sig**.
1: “*How anxious/worried do you feel about the course of your disease*?”	5.4 (2.1)	6.4 (2.3)	1.1 (2.0)	8.7	<0.01
2: “*How vulnerable do you feel because of your disease?”*	5.6 (2.2)	6.9 (2.5)	1.3 (1.9)	10.8	<0.01
3: “*How disoriented/confused do you feel about managing your disease*?”	4.6 (2.5)	5.8 (2.8)	1.2 (2.0)	8.9	<0.01
4: “*To what extent do you feel you have control over your disease/you are able to manage your disease*?”	6.2 (2.2)	5.7 (2.3)	−0.6 (1.9)	−4.5	<0.01
5: “*How sad/discouraged do you feel regarding your disease*?”	5.3 (2.7)	6.1 (2.7)	0.8 (1.8)	7.1	<0.01
6: “*How much energy have you invested in finding support and help in managing your disease*?”	5.3 (2.8)	5.3 (2.9)	−0.0 (1.8)	−0.0	0.97
7: “*How much energy have you invested in finding effective strategies to manage your disease*?”	6.2 (2.6)	6.1 (2.6)	−0.04 (1.8)	−0.4	0.69

No significant differences emerged between the two examined age groups (18–30 vs. 31–45 years) for the changes between “during” and “before” COVID-19 in self-reported perceptions about MS. Similarly, these changes did not significantly differ among subgroups regarding the other main sociodemographic characteristics or place of living (north/central/south regions of Italy). See Tables 1–5 in Appendix 2 for a detailed description of the results for each item.

### Changes in Care Management and Related Perceived Stress

Half of the respondents (*n* = 123) reported changes in MS management during the COVID-19 pandemic. Table 2 summarizes results regarding the main changes in the healthcare process, including medical, psychological, and physical treatment, as well as pharmacological therapies and re-organization of services (e.g., telemedicine). Mean levels of stress attributed to each specific MS management change are reported as well. Canceled or postponed medical visits or exams were listed as the most frequent changes, whereas changes in pharmacological treatment represented the most stressful change. The general mean score of stress related to the changes described above was 6.5 (SD = 3; 3rd quartile = 8).

**Table 2:** Changes in medical treatment due to the COVID-19 pandemic and the related perceived stress (*n* = 123^*^).

**Items**	***N***	**Perceived stress (Mean stress value)**
Modified or postponed pharmacological treatment	27	7.9
Canceled or postponed physical treatment (e.g., physiotherapy, rehabilitation)	9	7.2
Canceled or postponed medical visits or exams	78	6.6
Telehealth services	31	5.9
Canceled or postponed psychological treatment	19	5.7

* *Participants might select more than one change in medical treatment*.

### Needs/Preferences Regarding Psychological Support

Participants rated the importance of psychological support for young adults with MS in the aftermath of the emergency on a scale of 1 to 10 with a mean value of 8 (SD = 2; 3rd quartile = 10).

**Figure 2:**
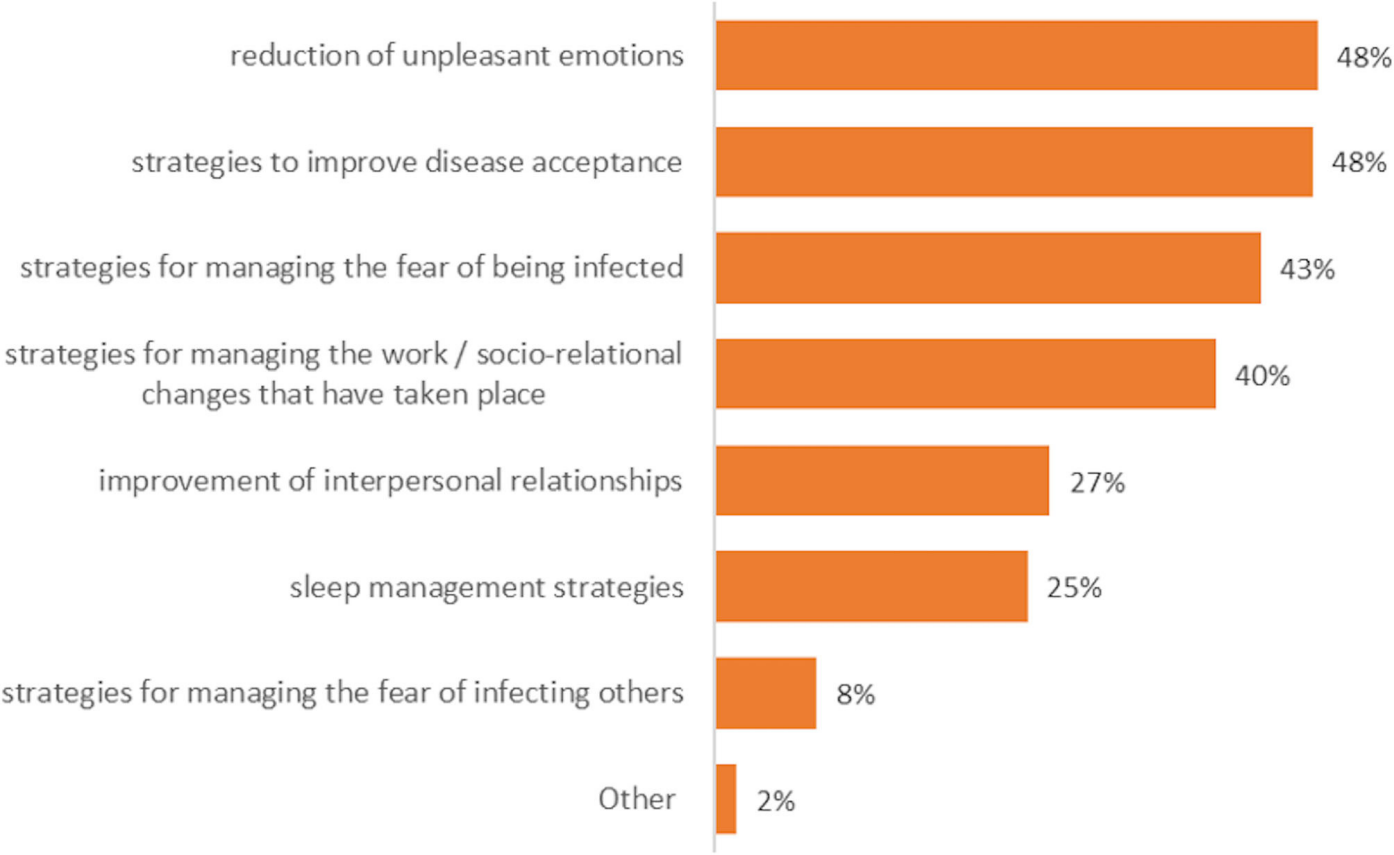
Topics that should be addressed in psychological support programs, by relevance.

As shown in Figure 2, among the respondents (*n* = 221), the majority reported that the psychological intervention should focus on the reduction of unpleasant emotions and on the promotion of strategies to improve MS acceptance, to manage the fear of being infected and the work/socio-relational changes due to the pandemic.

### Coping Strategies

Two hundred and eleven MS patients (85%) responded to the qualitative section of the survey, with six being excluded as they answered “*no strategy.”* Two hundred and five patients (83%) described coping strategies in dealing with pandemic-linked stress and were included in the following qualitative analysis. Twelve different types of coping strategies were indicated by respondents as useful to deal with the stress, with *social support, hobbies*, and *keeping oneself busy* being the most applied ones (see Table 3). It has to be noted that 52 respondents reported more than one coping strategy.

**Table 3:** Applied coping strategies during the time of the pandemic, including examples and frequencies (*n* = 205).

**Type of coping strategy**	**Description**	**Example**	**Number of quotes**
Social support	Turning to friends, the partner, family members, children, or psychosocial support^*^, using also different types of telecommunication	• “*Cultivating relationships, primarily with my partner, but also with friends and family (via video calls and messages)”* • “*Talking about my fears”*	44
Hobbies	Finding new hobbies and/or revisiting old ones, including, for example, reading, gardening, painting, English course, cooking	• “*Starting with new hobbies”* • “*Devoting myself to the things that I like but that before I had no time and way to do”*	42
Keeping oneself busy	Efforts to stay active and be occupied, in particular with mental activity and work/studying	• “*Focusing on work”* • “*Doing and learning new things at home that allowed me at that time not to think about multiple sclerosis and various difficulties”*	39
Positive thinking	Efforts to think positively, including also positive reappraisal (i.e., attempts to re-construe stressful events as valuable or useful)	• “*Taking advantage of uncomfortable situations to create something good”* • “*Re-evaluating loneliness as an opportunity to feel good with yourself”*	29
Following rules/recommendations to prevent the transmission of COVID-19	Behaviors that follow the prevention and containment measures taken by the government and/or the recommendations given by healthcare organizations	• “*Limiting personal movement and contact with others as much as possible”* • “*Taking the necessary precautions without panicking”*	24
Physical activity	Efforts to be physically active, either at home or outside	• “*Doing physical activity at home or even better outdoors in the garden to enjoy also the sunlight”* • “*Keeping active, doing exercises”*	22
Meditation/relaxation	Efforts to relax one's stressed mind, applying also specific relaxation techniques, such as meditation and breathing	• “*Having done mindfulness exercises was very useful”* • “*Thinking of these days as an opportunity to relax”*	19
Patience/acceptance	Efforts to accept or tolerate one's circumstances without becoming annoyed or upset	• “*Take the situation with a profound sense of calm”* • “*Taking life day by day”*	18
Experiential avoidance	“Attempts to avoid thoughts, feelings, memories, physical sensations, and other internal experiences even when doing so creates harm in the long-run,” (38) including experiences linked to the pandemic	• “*Avoiding to think about risk”* • “*Thinking as little as possible about reality”* • “*Avoiding thinking”*	15
Choosing sources of information carefully	Efforts to stay informed while being selective about news sources	• “*Informing oneself by reading reliable sources”* • “*Contacting the AISM* (*i.e., Italian Association of Multiple Sclerosis) to receive adequate information”*	9
Preserving routines	Endeavors to maintain one's pre-pandemic daily routine	• “*Keeping a routine with daily goals”* • “*Maintaining the pre-COVID routine”*	8
Setting/changing priorities	Endeavors to set new objectives and priorities and reassess one's set of priorities acquired prior to the pandemic	• “*Rearranging time and priorities”* • “*Considering it as opportunity to have time to restructure priorities and reorganize the day, doing what I really like to do”*	6

* *This category includes two quotes regarding psychosocial support received by psychological services*.

Table 6 in the Appendix 2 reported percentages of participants without a worsening of MS perceptions (i.e., emotions and illness perceptions) for each coping strategy. Social support, hobbies, and keeping oneself busy, which are the three strategies most frequently mentioned as effective by participants, seem to be associated to lower percentages of worsening in terms of sadness and disorientation.

## Discussion

This cross-sectional study has been conducted in the aftermath of the first wave of COVID-19 emergency in Italy. Findings allowed us to make a picture of an understudied population, such as the one of young adults with MS, regarding the psychological impact of the pandemic in terms of MS perceptions, changes in MS management, and consequent related perceived stress. Moreover, the main coping strategies evaluated to be useful by young adults with MS in dealing with the emergency and their perspective on the relevance of psychological support have been explored.

As regards the first aim of the current study, a higher level of anxiety, sadness, and confusion about MS emerged during the pandemic. Although the focus of the survey was on emotions regarding MS, results seem to confirm the negative impact of the pandemic on individuals' mental health, as already suggested by recent studies in the general population (24–26). As regards young adults, Liu et al. confirmed symptoms of depression, anxiety, post-traumatic stress disorder, loneliness, and poor resilience in adults under 30 years (39). Looking at the whole Italian context, among the general population, the young adult subgroup seems to be particularly vulnerable to the COVID-19 pandemic, as reported by Forte et al. who showed a greater negative psychological impact on people under 50 years (25).

However, considering the recent publications focusing on the psychological impact of COVID-19 pandemic in the MS population, results appear to be more controversial. Indeed, a study on patients with MS in a southern region of Italy indicated no increase in anxiety and depression and also some improvements on quality of life during the first wave of the pandemic (33), whereas other authors showed a high level of anxiety (29, 32). However, it has to be noted that, even if containment and control measures equally applied to all Italian regions during the period of the current study, the risk of contracting COVID-19 varied across Italy, and, as recognized by the Capuano et al., their contrasting results might be explained also by the fact that during the first wave, the southern regions of Italy were much less affected by the COVID-19 epidemic (33).

However, to consider the national differences in pandemic spread, which may have led to varying degrees of perceived risk and uncertainty among respondents, depending on area of residence, and, additionally, the potential differences in organization and delivery of MS health services, which might be different across the various MS centers, a comparison of changes in MS perceptions among survey participants living in the northern, center, or southern regions has been conducted, resulting in no differences. Similar to our results, the recent study of Forte et al. (25) assessing different measures of psychological impact found significant differences between the north, center, and south of Italy only for sleep disturbances.

Moreover, a similar worsening in emotions and illness perceptions linked to MS emerged when considering both young adults under 30 years or the ones in the 31–45 age range and other sociodemographic characteristics.

The increase of anxiety, sadness, and confusion is consistent with the theme of uncertainty, which might be considered a common feeling that characterized the experience of people during the pandemic, due to the novelty of the emergency, severe symptoms, high mortality rates, lack of cure, the severe consequences for daily life and society, and the highly unpredictable future course of the pandemic (25, 40). Uncertainty is a familiar psychological construct for people who have to live with chronic illness. Indeed, due to the complexity and unpredictability of clinical course and potential treatment side effects, people with MS have to accept in their adaptation to illness and chronicity that not all the aspects of their health can be controlled and foreseen (41–43). In line with this evidence, our results suggest that the public health emergency has negatively influenced MS perceptions for young adults with MS, leading to an increased sense of vulnerability and lack of control over MS.

As regards our second aim, half of the respondents highlighted at least one change in the MS management due to the COVID-19 pandemic. Although respondents indicated an overall moderate level of perceived stress, 44% considered these changes as severe (i.e., 8–10 on the 10-point Likert scale). The high relevance of MS management changes is consistent with a previous research, highlighting the fact that patients with MS in Saudi Arabia reported a significant impact of the pandemic on their MS healthcare, in particular due to fear of being infected (44). Although canceled or postponed medical visits or exams have been, according to the participants' perspective, the most frequent change in MS management, the modified or postponed pharmacological treatment had the worst impact on perceived stress. Though we did not explore this relationship in our analysis, we might hypothesize that changes in routine clinical procedures, a reduced number of encounters with healthcare professionals, and the postponement of care and the associated perceived stress might have also contributed to uncertainty and thus the reduced level of perceived illness control and increased vulnerability perception. To support this consideration, a recent Serbian study reported the impossibility to go to hospital as usual, the difficulties in drug availability, and the worsening of the MS status in case of contagion as the main concerns regarding relapsing–remitting MS during the pandemic outbreak. During the pandemic, the use of telemedicine and digital services has been widely extended to compensate for the reduction of services provided in person. As previously suggested, telemedicine should be offered in order to effectively respond to the needs of MS patients in times of emergency, such as the COVID-19 pandemic (21, 23, 45). Moreover, considering the high use of web and mobile applications by young adults, e-health resources, aiming to provide strategies and support in dealing with stress and emotions linked to the pandemic, might be especially useful. Indeed, as regards our third aim, almost 40% of the respondents rated as extremely important (i.e., 10 on Likert scale) the opportunity to receive psychological support, stressing not only the support in dealing with emotions and MS acceptance in general but also in managing the fear of being infected.

Looking at the last explorative aim, our respondents seemed to adopt a wide range of coping strategies to deal with the stress linked to the COVID-19 emergency. Overall, looking to the variety of coping strategies, the most frequent strategies evaluated as useful in patients' eyes were social support, hobbies, and keeping oneself busy. Results are quite different from what was observed in a previous study where active coping and religion were the strategies most frequently adopted in order to face COVID-19 (31). Conversely, the relevance of those coping strategies, which emerged in our study, was consistent with other results reported during the COVID-19 pandemic and, more in general, in the chronic disease field, as discussed in the section below.

The evaluation of social support as one of the main coping strategy in dealing with the pandemic is coherent with the consideration that, in general, perceived social support and positive relationships are associated with better MS adjustment (41). Consistently, in the other Italian study mentioned above, authors highlighted that patients during the pandemic might have experienced a higher social support (e.g., more opportunity to spend time with family members) that could have been associated to the absence of worsening in depressive symptoms (33). Similarly, a recent survey assessing US young adults during the COVID-19 pandemic reported that family support is associated with low levels of psychological symptoms (39). The category “social support” contained not only respondents' quotes regarding the support received by others but also the support they provided to relevant people during the pandemic, thus suggesting that in challenging or even threatening situations, connection and empathy among relatives and friends might play a significant role in protecting from perceived stress.

Interestingly, among the most frequently useful coping strategies, we also found pleasant and meaningful activities in everyday life that mostly rely on personal internal resources (i.e., hobbies), suggesting the beneficial power of engaging in value-consistent actions that stimulate creativity (e.g., cooking, painting) and personal growth (e.g., reading). Similarly, physical activities and meditation/relaxation also emerged as relevant strategies for respondents' personal well-being.

“Keeping oneself busy” was also frequently considered as effective by young adults with MS. A similar result emerged in Umucu and Lee (46), who highlighted self-distraction with other activities as one of the most frequent coping strategies with COVID-19 among participants with chronic conditions and disability. As reported in a previous study in the context of chronicity, this strategy might have a controversial role in terms of adaptiveness (47). In general, we might assume that if it is applied to deny stress and avoid any stressors, it can be maladaptive for emotion regulation. In contrast, if it is used to mitigate uncertainty and fear linked to COVID-19 in the short period, it might help to preserve a sense of self-efficacy and control in daily life.

Similar to the study by Umucu et al. who reported the “tendency to deny the reality of COVID-19” as the least commonly used strategy, in our sample, “experimental avoidance” also emerged only in a small number of quotes (46).

Although the survey was not aimed to make a diagnosis of psychiatric disorders or identify patients with high risk of scarce adaptation to MS, the detected significant negative psychological impact indicates that this aspect has to become one of the priorities of clinical centers when restarting routine healthcare after the emergency phase and during the further wave(s) of the pandemic. Indeed, unfortunately in Italy, at the moment in which this paper is under revision, a second wave of the pandemic is occurring with healthcare services being newly affected by reduced visits and changes in MS management. Considering changes in MS perception and reducing stress linked to changes in MS management is a priority for healthcare professionals working in MS field, not only because it is strictly linked to patients' global well-being but also because it has implications for patient empowerment in illness management. Indeed, emotional dimensions interact with risk appraisal, rational thinking, and decision-making, key elements that influence health decisions and proactive behaviors in chronic illness (48). Considering the increased level of vulnerability perception and reduced control over the disease, and the continuous uncertainty linked to the further actual spread of the pandemic, psychological interventions, also through telemedicine, should reinforce the therapeutic alliance and continuity of care through empathic and supportive listening. Specifically, as part of a larger project ESPRIMO, results derived from this explorative survey will contribute to inform the development of the specific ESPRIMO biopsychosocial intervention aiming to promote resilience and health-related quality of life in young adults with MS and, more broadly, enhance patients' adaptation to MS. Indeed, the ESPRIMO intervention should not avoid to also address the potentially long-lasting negative psychological impact and MS management changes caused by the COVID-19 pandemic on this particularly vulnerable clinical population. This also considers that the target population is in the age range of onset of the disease, a period especially difficult for the psychosocial adjustment to the disease (9). On the basis of the current results, the psychological component of the intervention should also foster strategies to better deal with the fear of being at risk of being contagious for COVID-19 and to reduce the sense of vulnerability and the stress linked to the uncertainty and to changes in MS management and everyday life. The group modalities will characterize the intervention and should favor a shared expression of the COVID-19 experience to foster a sense of acceptance, belonging, and security and potential releases of psychological impact of the pandemic together with the interpersonal learning on useful coping strategies.

### Strengths and Limitations

Over the past months, several studies have provided important information on the psychological impact of the COVID-19 pandemic on the general population, healthcare professionals, and some clinical populations, and, more recently, some articles have also explored its impact on MS populations. However, our study presents some novelty aspects that make it unique: exploring the negative effects of the current pandemic specifically for young adults with MS; focusing on the emotions, perceptions, and commitment specifically linked to MS; and using both quantitative and qualitative methods.

Recruiting adults with MS within the ages of 18 to 45 allowed us to explore a group that is particularly vulnerable in terms of adjustment to live with the disease, which is often diagnosed in this age range.

The open qualitative exploration of patients' perspective on self-perceived functional coping enabled us to capture a wide, genuine, and heterogeneous description of all the coping strategies applied during the pandemic by patients, in order to face the risk of infection and their disease. This is in line with our adoption of a resource-based approach oriented to raising and valuing patients' awareness of personal strategies. As previously highlighted, this qualitative approach allowed us to explore an aspect still little investigated, especially in the period when we performed the survey, which was during the first wave of the pandemic when the first priority was to detect emerging psychological needs in the population subject of our clinical interest not making a priori assumption on their coping style. By giving to participants the possibility of reporting their own strategies, we recognize the patients' role as experts; patients feel reassured and motivated in this way and therefore free to report about their own experience, without the fear of saying “something wrong” (49). Future psycho-educational or supporting interventions, based on suggestions provided by peers on the basis of their own real experiences during the first wave of COVID-19, might represent a particularly valuable resource for patients facing further waves of the pandemic.

Furthermore, using online modality for our survey was appropriate considering the explorative nature of the study but presents both limitations and strengths. As regards limitations, the fulfillment of inclusion criteria was self-evaluated by participants, thus limiting the control on the sampling and potentially the accuracy of the answers. As regards strengths, using online modality guaranteed safety in the aftermath of the COVID-19 pandemic; had the ability to reach a larger pool of potential participants within a shorter period of time, reducing costs and improving efficiency of data collection and management; allowed the recruitment of patients from the whole of Italy, including patients otherwise difficult to approach; and provided improved comfort and sense of control for participants (50, 51). Moreover, it has to be considered that social media represented an especially powerful tool to recruit our specific sample, since it is known to be a very popular channel of interaction for young adults (52). As regards the second aspect, it has to be considered that by advertising the survey among online MS groups, we reached mainly patients already involved in MS management and probably missed more isolated patients, who might represent also the most vulnerable group. Moreover, in terms of generalizability, considering that almost all the respondents reported a diagnosis of relapsing–remitting MS, our study did not sufficiently capture the psychological impact and needs of patients with different types of MS during COVID-19 emergency. Moreover, given that participation was voluntary, it might be assumed that patients more interested in psychological topics were more likely to participate, thus leading to a potential selection bias.

A strength lies in the comparison of patients' emotions regarding MS, illness perception, and commitment to deal with MS before and during the pandemic. Although a specific time frame is difficult to define and might vary subjectively since the spread of COVID-19 varied across Italian regions, the survey has been conducted in the aftermath of the first wave of the pandemic, and in answering, patients refer to a period characterized by major quarantine policies in place (e.g., severe restrictions of movement, reduced outpatients visits in most of the public and private hospitals, complete or partial shutdowns of economic and social activities) and a widespread perception of high risk due to the large numbers of reported cases and deaths. Although this methodology has been previously used in another Italian paper on COVID-19 (25), it must be noted that participants' retrospective self-reports might be influenced by recall bias, thus potentially influencing the perception of the participants and the validity of our findings. Therefore, the lack of a proper longitudinal design for evaluating the psychological impact represents one of the main limitations of the study. Moreover, we preferred to create an *ad hoc* scale for this specific aim of the study, which showed high reliability. However, the scale was not validated.

The fact that our sample covered all the Italian regions represents an additional strength, as previously reported being able to collect data from areas with different degrees of transmission. Indeed, the only previous study that considered the psychological impact of the pandemic on patients with MS in Italy was based on a relatively small sample size of patients living in a specific region in the south of Italy. However, regarding the sociodemographic and clinical information explored in the survey, some weakness has to be highlighted too; in particular, the exploration of gender was limited to just a low percentage of our participants. Moreover, we did not assess and consider if participants or their family members were actually exposed to the virus and got infected. Therefore, future studies are needed in order to deepen the role played by sociodemographic and clinical characteristics on the psychological impact of COVID-19 within chronicity.

## Conclusions

Tackling the psychological impact of COVID-19 international emergency and of the consequent changes in healthcare organizations aimed to reduce the risk of disease transmission should be a top priority for MS clinical centers that are striving for continuous improvement. Psychological support programs tailored to patients' individual psychological needs and preferences would help to strengthen the therapeutic alliance that has been threatened by the infection prevention measures imposed during the pandemic and to foster patients' capacity for adaptation and “bouncing back” in the face of significant sources of stress [i.e., resilience (53)] such as the COVID-19 experience.

## Data Availability Statement

The datasets presented in this article are available from the corresponding author on reasonable request.

## Ethics Statement

The studies involving human participants were reviewed and approved by CESC: Ethical Committee of the Verona Hospital. The patients/participants provided their informed consent to participate in this study.

## Author Contributions

MR and VD conceived the study and the study design and drafted the manuscript. MM performed the statistical analysis. MR, VD, and AGh performed the qualitative analysis. IB, FG, AGh, and AGa contributed with data collection and relevant contents to the drafted manuscript. All authors reviewed and revised the manuscript.

## Conflict of Interest

AGa reports personal fees and non-financial support from Biogen, Merck, grants from Cesare Serono Foundation, Eurimmun, and Cariverona Foundation, outside the submitted work. The remaining authors declare that the research was conducted in the absence of any commercial or financial relationships that could be construed as a potential conflict of interest.
