# Supplementary material for: Insights for Fostering Resilience in Young Adults With Multiple Sclerosis in the Aftermath of the COVID-19 Emergency: An Italian Survey
Source: Front Psychiatry. 2021 Feb 22;11:588275. doi: 10.3389/fpsyt.2020.588275 (PMC7938709; doi:10.3389/fpsyt.2020.588275)
Supplement: Supplementary file 1 [file Data_Sheet_1.docx]

**The following questions are aimed at understanding the impact of the Covid-19 health emergency on experiences (emotions, concerns, perceptions) and the management of the disease. Furthermore, specific needs will be explored that can guide future interventions.**

**Section 1: Emotions, illness perception, commitment to deal with MS DURING COVID-19 EMERGENCY**

**B01)** How much do you **feel worried/anxious** about the course of your disease?

*Before the COVID-19 emergency*

*Not at all* 1 2 3 4 5 6 7 8 9 10 *Very much*

*During the COVID-19 emergency*

*Not at all* 1 2 3 4 5 6 7 8 9 10 *Very much*

**B02)** How much do you feel **vulnerable** because of your disease?

*Before the COVID-19 emergency*

*Not at all* 1 2 3 4 5 6 7 8 9 10 *Very much*

*During the COVID-19 emergency*

*Not at all* 1 2 3 4 5 6 7 8 9 10 *Very much*

**B03)** How much do you feel **confused/disoriented** about the course of your disease?

*Before the COVID-19 emergency*

*Not at all* 1 2 3 4 5 6 7 8 9 10 *Very much*

*During the COVID-19 emergency*

*Not at all* 1 2 3 4 5 6 7 8 9 10 *Very much*

**B04)** How much do you **feel in control/able to manage** your disease?

*Before the COVID-19 emergency*

*Not at all* 1 2 3 4 5 6 7 8 9 10 *Very much*

*During the COVID-19 emergency*

*Not at all* 1 2 3 4 5 6 7 8 9 10 *Very much*

**B05)** How much do you feel **sad/ discouraged** regarding your disease?

*Before the COVID-19 emergency*

*Not at all* 1 2 3 4 5 6 7 8 9 10 *Very much*

*During the COVID-19 emergency*

*Not at all* 1 2 3 4 5 6 7 8 9 10 *Very much*

**B06)** How many energies do you invested in searching for **support and help** for the management of your disease?

*Before the COVID-19 emergency*

*Not at all* 1 2 3 4 5 6 7 8 9 10 *Very much*

*During the COVID-19 emergency*

*Not at all* 1 2 3 4 5 6 7 8 9 10 *Very much*

**B07)** How many energies do you invested in searching for **effective ways** to manage your disease?

*Before the COVID-19 emergency*

*Not at all* 1 2 3 4 5 6 7 8 9 10 *Very much*

*During the COVID-19 emergency*

*Not at all* 1 2 3 4 5 6 7 8 9 10 *Very much*

**Section 2: MS management changes and perceived stress regarding changes DURING COVID-19 EMERGENCY**

**B08)** In recent months, in relation to the covid-19 emergency, the management of your disease has changed?

🞎Yes

🞎No

**B09)** If so, what were the main changes?

🞎 Cancellation or postponement of the scheduled visit or exams with my referring physician

🞎 Cancellation or postponement of the scheduled psychological visit with my referring psychologist

🞎 Reorganization of clinical services in telematic mode (telephone, skype)

🞎 Modified or postponed pharmacological treatment

🞎 Others (please specify) ______________________

**B10)** How much **distress/discomfort** the change caused?

*Not at all* 1 2 3 4 5 6 7 8 9 10 *Very much*

**Section 3: Needs and resources related to the covid-19 emergency**

**B11)** In relation to the Covid19 emergency, how important would it be to implement a **psychological support service** for stress management available to young patients with multiple sclerosis

*Not important* 1 2 3 4 5 6 7 8 9 10 *Very important*

**B12)** Which areas should it focus on (indicate a maximum of 3)?

🞎 Lowering of unpleasant emotions

🞎 Improvement of interpersonal relationships

🞎 Sleep management strategies

🞎 Strategies for managing the fear of being infected

🞎 Strategies for managing the fear of infecting other people

🞎 Strategies to improve acceptance of the disease

🞎 Strategies for managing work/socio-relational changes that have occurred

🞎 Other aspect(s) (please specify) ______________________

**Section 4: Psychological resources during to the covid-19 emergency**

**B13)** Please complete the following sentence: **“The strategy that has proven most useful for getting through this pandemic period has been…”**

**B14)** Please complete the following sentence: “**the thought that has helped me the most to get through this period of quarantine has been…”**

**B15)** Please complete the following sentence: “**The most precious thing that quarantine has taught me is** …”
