# Supplementary material for: Insights for Fostering Resilience in Young Adults With Multiple Sclerosis in the Aftermath of the COVID-19 Emergency: An Italian Survey
Source: Front Psychiatry. 2021 Feb 22;11:588275. doi: 10.3389/fpsyt.2020.588275 (PMC7938709; doi:10.3389/fpsyt.2020.588275)
Supplement: Supplementary file 2 [file Data_Sheet_2.docx]

**Appendix 2**

Table 1-5. Comparison of changes between “before” and “during” covid-19 across the main socio-demographic characteristics:

Table 1 “How anxious/worried do you feel about the course of your disease?”

|  | N | Mean Δ | Sd Δ | t/ANOVA | p |
| --- | --- | --- | --- | --- | --- |
| *Age* |  |  |  | 0.51 | 0.61 |
| 18-30 | 108 | 1.16 | 1.80 |  |  |
| 31-45 | 139 | 1.03 | 2.07 |  |  |
| *Gender** |  |  |  | 1.18 | 0.25 |
| F | 26 | 1.61 | 1.24 |  |  |
| M | 18 | 1.11 | 1.60 |  |  |
| *Educational status* |  |  |  | 0.32 | 0.75 |
| Without Academic Degree | 139 | 1.05 | 2.03 |  |  |
| With Undergraduate/Graduate Degree | 108 | 1.13 | 1.87 |  |  |
| *Occupation* |  |  |  | 0.06 | 0.95 |
| Yes | 137 | 1.09 | 1.82 |  |  |
| No | 100 | 1.11 | 2.03 |  |  |
| *Married/in a relationship* |  |  |  | 1.61 | 0.11 |
| Yes | 113 | 0.87 | 2.06 |  |  |
| No | 134 | 1.27 | 1.85 |  |  |
| *Zone* |  |  |  | 0.74 | 0.48 |
| North | 117 | 0.93 | 2.02 |  |  |
| Centre | 47 | 1.30 | 1.65 |  |  |
| South | 83 | 1.18 | 2.03 |  |  |

* the analysis has been performed just on a subsample of about 30% of the participants.

Table 2 *“How vulnerable do you feel because of your disease?”*

|  | N | Mean Δ | Sd Δ | t/ANOVA | p |
| --- | --- | --- | --- | --- | --- |
| *Age* |  |  |  | 1.16 | 0.25 |
| 18-30 | 108 | 1.12 | 1.67 |  |  |
| 31-45 | 139 | 1.40 | 1.99 |  |  |
| *Gender** |  |  |  | 1.42 | 0.16 |
| F | 26 | 1.69 | 2.02 |  |  |
| M | 18 | 089 | 1.57 |  |  |
| *Educational status* |  |  |  | 1.33 | 1.18 |
| Without Academic Degree | 139 | 1.14 | 1.92 |  |  |
| With Undergraduate/Graduate Degree | 108 | 1.45 | 1.77 |  |  |
| *Occupation* |  |  |  | 1.14 | 0.26 |
| Yes | 137 | 1.44 | 1.94 |  |  |
| No | 100 | 1.16 | 1.73 |  |  |
| *Married/in a relationship* |  |  |  | 0.69 | 0.49 |
| Yes | 113 | 1.19 | 1.89 |  |  |
| No | 134 | 1.35 | 1.83 |  |  |
| *Zone* |  |  |  | 1.27 | 0.28 |
| North | 117 | 1.11 | 1.75 |  |  |
| Centre | 47 | 1.62 | 2.08 |  |  |
| South | 83 | 1.31 | 1.87 |  |  |

* the analysis has been performed just on a subsample of about 30% of the participants.

Table 3 “*How disoriented/confused do you feel about managing your disease*?”

|  | N | Mean Δ | Sd Δ | t/ANOVA | p |
| --- | --- | --- | --- | --- | --- |
| *Age* |  |  |  | 1.52 | 0.13 |
| 18-30 | 108 | 0.94 | 1.76 |  |  |
| 31-45 | 139 | 1.33 | 2.21 |  |  |
| *Gender** |  |  |  | 1.34 | 0.19 |
| F | 26 | 1.58 | 2.28 |  |  |
| M | 18 | 0.78 | 1.31 |  |  |
| *Educational status* |  |  |  | 0.12 | 0.90 |
| Without Academic Degree | 139 | 1.14 | 1.95 |  |  |
| With Undergraduate/Graduate Degree | 108 | 1.18 | 2.16 |  |  |
| *Occupation* |  |  |  | 0.80 | 0.42 |
| Yes | 137 | 1.09 | 1.96 |  |  |
| No | 100 | 1.30 | 2.07 |  |  |
| *Married/in a relationship* |  |  |  | 1.63 | 0.11 |
| Yes | 113 | 0.93 | 2.01 |  |  |
| No | 134 | 1.35 | 2.05 |  |  |
| *Zone* |  |  |  | 1.84 | 0.16 |
| North | 117 | 0.91 | 1.90 |  |  |
| Centre | 47 | 1.21 | 2.14 |  |  |
| South | 83 | 1.47 | 2.15 |  |  |

* the analysis has been performed just on a subsample of about 30% of the participants.

Table 4 “*To what extent do you feel you have control over your disease/you are able to manage your disease*?”

|  | N | Mean Δ | Sd Δ | t/ANOVA | p |
| --- | --- | --- | --- | --- | --- |
| *Age* |  |  |  | 0.69 | 0.49 |
| 18-30 | 108 | -0.46 | 1.69 |  |  |
| 31-45 | 139 | -0.63 | 2.10 |  |  |
| *Gender** |  |  |  | 1.02 | 0.31 |
| F | 26 | -1.08 | 2.24 |  |  |
| M | 18 | -0.50 | 0.99 |  |  |
| *Educational status* |  |  |  | 1.51 | 0.13 |
| Without Academic Degree | 139 | -0.40 | 1.80 |  |  |
| With Undergraduate/Graduate Degree | 108 | -0.77 | 2.0 |  |  |
| *Occupation* |  |  |  | 0.01 | 0.99 |
| Yes | 137 | -0.52 | 1.87 |  |  |
| No | 100 | -0.52 | 2.02 |  |  |
| *Married/in a relationship* |  |  |  | 1.33 | 0.18 |
| Yes | 113 | -0.38 | 1.94 |  |  |
| No | 134 | -0.71 | 1.91 |  |  |
| *Zone* |  |  |  | 0.01 | 0.99 |
| North | 117 | -0.57 | 1.84 |  |  |
| Centre | 47 | -0.53 | 1.87 |  |  |
| South | 83 | -0.55 | 2.10 |  |  |

* the analysis has been performed just on a subsample of about 30% of the participants.

Table 5 “*How sad/discouraged do you feel regarding your disease*?”

|  | N | Mean Δ | Sd Δ | t/ANOVA | p |
| --- | --- | --- | --- | --- | --- |
| *Age* |  |  |  | 1.43 | 0.15 |
| 18-30 | 108 | 0.64 | 1.72 |  |  |
| 31-45 | 139 | 0.97 | 1.88 |  |  |
| *Gender* |  |  |  | 1.04 | 0.30 |
| F | 26 | 1.08 | 1.85 |  |  |
| M | 18 | 0.56 | 1.25 |  |  |
| *Educational status* |  |  |  | 1.12 | 0.27 |
| Without Academic Degree | 139 | 0.71 | 1.80 |  |  |
| With Undergraduate/Graduate Degree | 108 | 0.97 | 1.83 |  |  |
| *Occupation* |  |  |  | 0.35 | 0.73 |
| Yes | 137 | 0.82 | 1.82 |  |  |
| No | 100 | 0.90 | 1.74 |  |  |
| *Married/in a relationship* |  |  |  | 0.09 | 0.93 |
| Yes | 113 | 0.81 | 1.84 |  |  |
| No | 134 | 0.84 | 1.80 |  |  |
| *Zone* |  |  |  | 0.19 | 0.83 |
| North | 117 | 0.75 | 1.72 |  |  |
| Centre | 47 | 0.91 | 2.03 |  |  |
| South | 83 | 0.88 | 1.84 |  |  |

* the analysis has been performed just on a subsample of about 30% of the participants.

Table 6. Percentages of participants without a worsening of MS perceptions (i.e., emotions and illness perceptions) for each coping strategy.

| **Type of coping strategy** | **Number of quotes** | **Item 1**  **Anxiety** | **Item 2**  **vulnerability** | **Item 3**  **Disorientation** | **Item 4**  **Control^*^** | **Item 5**  **Sadness** |
| --- | --- | --- | --- | --- | --- | --- |
| Social support | 44 | 41% | 32% | 52% | 16% | 57% |
| Hobbies | 42 | 41% | 38% | 52% | 14% | 67% |
| Keeping oneself busy | 39 | 38% | 36% | 54% | 10% | 64% |
| Positive thinking | 29 | 41% | 38% | 62% | 3% | 59% |
| Following rules/recommendations to prevent the transmission of COVID-19 | 24 | 38% | 29% | 46% | 13% | 67% |
| Physical activity | 22 | 32% | 23% | 64% | 14% | 55% |
| Meditation/relaxation | 19 | 32% | 16% | 47% | 16% | 58% |
| Patience/acceptance | 18 | 33% | 33% | 56% | 6% | 61% |
| Experiential avoidance | 15 | 47% | 33% | 60% | 13% | 27% |
| Choosing sources of information carefully | 9 | 44% | 33% | 67% | 11% | 67% |
| Preserving routines | 8 | 38% | 38% | 50% | 0% | 50% |
| Setting/changing priorities | 6 | 67% | 50% | 50% | 33% | 67% |

The percentages are referred to the number of responders without a worsening of MS perceptions (i.e., delta ≤ 0) on the total responders for each specific coping strategy. For example, 44 responders used social support as effective strategy, among these 18 responders (41%) resulted without a worsening of anxiety linked to MS. *The delta of this item has been inverted (see Table 1) in order to express the percentage of responders without a worsening of MS control.
